# Left and Right Ventricular Morphology, Function and Myocardial Deformation in Children with Left Ventricular Non-Compaction Cardiomyopathy: A Case-Control Cardiovascular Magnetic Resonance Study

**DOI:** 10.3390/jcm11041104

**Published:** 2022-02-19

**Authors:** Jędrzej Sarnecki, Agata Paszkowska, Joanna Petryka-Mazurkiewicz, Agata Kubik, Janusz Feber, Elżbieta Jurkiewicz, Lidia Ziółkowska

**Affiliations:** 1 Department of Diagnostic Imaging, The Children’s Memorial Health Institute, 04-730 Warsaw, Poland; j.sarnecki@ipczd.pl (J.S.); e.jurkiewicz@ipczd.pl (E.J.); 2 Department of Cardiology, The Children’s Memorial Health Institute, 04-730 Warsaw, Poland; a.paszkowska@ipczd.pl; 3 Department of Coronary and Structural Heart Diseases, National Institute of Cardiology, 04-628 Warsaw, Poland; j.petryka@ikard.pl; 4 Magnetic Resonance Unit, National Institute of Cardiology, 04-628 Warsaw, Poland; akubik@ikard.pl; 5 Division of Nephrology, Children’s Hospital of East Ontario, University of Ottawa, Ottawa, ON K1H 8L1, Canada; jfeber@cheo.on.ca

**Keywords:** left ventricular non-compaction, children, cardiovascular magnetic resonance, strain, tissue-tracking, late gadolinium enhancement

## Abstract

*Background*: Left ventricular non-compaction (LVNC) is a rare cardiomyopathy typically involving the left ventricle (LV); however, the right ventricle (RV) can also be affected. This case-control study aimed to assess the morphology and function of LV and RV in children with LVNC. *Methods*: Sixteen children (13 ± 3 years, six girls) with LVNC were compared with 16 sex- and age-matched controls. LV and RV morphology and function were evaluated in cardiovascular magnetic resonance (CMR) studies. Additionally, LV and RV global radial (GRS), circumferential (GCS), and longitudinal strain (GLS) were assessed using tissue-tracking analysis. *Results*: Patients with LVNC did not differ from the healthy controls in terms of age, height, weight, and body surface area (BSA). In total, 4/16 subjects with LVNC had mid-wall late gadolinium enhancement (LGE). Compared to the control group, patients with LVNC had higher end-diastolic volume (EDV) indexed for body surface area (BSA), lower ejection fraction (EF), and lower LV strain parameters (all *p* < 0.05). Children with LVNC also presented with thicker RV apical trabeculation, whereas there were no differences in RV EF and EDV/BSA between the groups. Nevertheless, children with LVNC had impaired RV GRS and GCS (both *p* < 0.05). *Conclusions*: LVNC in pediatric patients is associated with LV enlargement and impaired LV systolic function. Additionally, children with LVNC have increased RV trabeculations and subclinical impairment of RV myocardial deformation.

## 1. Introduction

Left ventricular non-compaction (LVNC) is a rare, yet gradually more recognized myocardial pathology. It constitutes the third most prevalent primary cardiomyopathy in the pediatric population, after dilated and hypertrophic cardiomyopathies, and accounts for nearly 10% of cardiomyopathy cases in children [1,2]. LVNC may be an isolated disorder or present with other congenital heart defects or neuromuscular diseases [3,4]. Multiple genetic mutations associated with the condition have been discovered, and a family history of cardiomyopathies is often found in affected individuals [5]. LVNC has a very heterogeneous clinical course. Patients can be completely asymptomatic, paucisymptomatic, or present with arrhythmias, thromboembolic episodes, or congestive heart failure, sometimes leading to heart transplantation or death [2,6,7,8,9]. In the pediatric population, transplantation-free survival was reported to be as low as 45% at 15 years after presentation, which is significantly poorer than in adults, probably due to high mortality among affected infants [2].

LVNC typically involves the LV, which has a thin compacted myocardial layer and prominent trabeculations and intertrabecular recesses [3,4]. Nevertheless, the right ventricle (RV) can also be affected, and in adults with LVNC, RV dysfunction was shown to be associated with worse prognosis [10,11]. To date, the prevalence of increased RV trabeculation in children with LVNC was reported in several studies, with no data on RV volumes and function assessed using CMR in affected pediatric patients available [12,13,14].

CMR is the reference method for the non-invasive evaluation of ventricular mass, volume, and function, as well as for the characterization of the myocardium. Its capacity to provide accurate and reproducible information on cardiac morphology and function renders it the best currently available noninvasive diagnostic method for assessing both ventricles in children with LVNC [15,16]. Several analyses conducted in adults showed that increased LV EDV, decreased LV EF, and presence of LGE evaluated in CMR provide long-term prognostic information on cardiac event risk in patients with LVNC [17]. Fewer CMR studies on LVNC in children were conducted, with equivocal results on cardiac morphology and function [18,19,20].

This case-control study aimed to evaluate LV and RV morphology and function in pediatric patients with LVNC and to compare them with age- and sex-matched controls. In addition, we sought to assess the differences between children with LVNC, in whom LGE was detected, and the remaining LVNC patients without LGE.

## 2. Materials and Methods

### 2.1. Study Population

The study cohort consisted of 16 children aged 6–17 years, prospectively recruited among hospitalized patients with LVNC suspected on echocardiography and subsequently confirmed in CMR. LVNC was diagnosed using the Jacquier criteria, i.e., when trabeculated left ventricular mass (LVM) >20% of global LVM [21]. Children with associated congenital heart diseases were excluded from the study. Patients’ clinical symptoms such as chest pain, palpitations, syncope, thromboembolic events, and history of cardiomyopathies and current treatment were recorded. The 12-lead resting electrocardiography (ECG) and 24 h Holter ECG results were recorded. The New York Heart Association (NYHA) functional class was evaluated in all children. Genetic tests were performed in all children in the study group by analyzing a panel of 1000 genes using next-generation sequencing technology (NGS).

Sixteen sex- and age-matched individuals with normal morphological and functional CMR scans were identified by database search and included as the control group. The indication for the CMR scan in the control group included non-diagnostic echocardiographic examination; suspected structural abnormalities on echocardiography; syncope or chest pain with a low pre-scan probability of being cardiac in origin; and suspected, but excluded in 24-h ambulatory blood pressure monitoring, primary hypertension.

### 2.2. Cardiovascular Magnetic Resonance Imaging

CMR imaging was conducted using a 1.5 T whole-body magnetic resonance scanner (Magnetom AvantoFit, Siemens, Erlangen, Germany), with a dedicated cardiac phased-array coil and ECG gating. Based on the scout and reference scans, steady-state free precession (SSFP) cine images of the heart in the standard four-, three-, and two-chamber planes were acquired. The ventricles were also visualized in the short-axis plane view, from the base, parallel, and across the atrioventricular valves, to the apex of the heart using 12 contiguous slices. The imaging was performed at end-expiration using the following parameters: repetition time (TR)/echo time (TE) 42.5/1.2 ms, spatial resolution = 8 × 1.5 × 1.5 mm, flip angle 62°, minimum 25 phases per cardiac cycle. In LVNC patients, late gadolinium-enhanced imaging was performed 10–15 min after contrast agent administration (0.1 mmol/kg of gadobutrol (Gadovist, Bayer, Berlin, Germany)).

### 2.3. Cardiac Magnetic Resonance Imaging Analysis

The CMR studies were analyzed using CVi42 (Circle Cardiovascular Imaging, Calgary, AB, Canada) on a dedicated diagnostic workstation. To determine the left ventricular compacted mass (LVM), left ventricular and right ventricular end-diastolic volume (LV EDV, RV EDV), and ejection fraction (LV EF, RV EF), the end-diastolic and end-systolic phases were identified based on the long-axis and the midventricular short-axis scans. LV endocardial and epicardial borders and RV endocardial borders were automatically contoured in those phases and then manually corrected. The interventricular septum and the papillary muscles were included in the compacted LVM [22]. Additionally, to assess LV global mass, LV endocardial borders were manually drawn to include both papillary muscles and LV trabeculations [21]. The trabeculated (noncompacted) LVM was established by subtracting compacted LVM from global LVM, and then the ratio between the trabeculated and the global LVM was calculated [21]. The thickness of the compacted (C) and the non-compacted (NC) LV myocardial layers was measured in end-diastole on long-axis scans (excluding the 17th segment according to American Heart Association), and the highest NC/C ratio value was recorded [23]. On the four-chamber cine images, RV apical trabecular thickness (ATT) was measured from the RV apex to the trabecular trough [10]. In the same projection, RV C and NC layers of the middle segments of the ventricle’s lateral wall were measured, and based on the results, RV NC/C ratio was determined [10].

LV and RV EDV, compacted LVM, and trabeculated LVM were indexed for BSA, determined using the Du Bois formula $BSA\;\left(m^{2}\right)=0.007184\times weight{\left(\mathrm{kg}\right)}^{0.425}\times height\;{\left(\mathrm{cm}\right)}^{0.725}$) [24]. To identify morphological abnormalities, LVM, LV EDV, and RV EDV were compared against recently published, multicenter CMR normative values for children and adolescents, which were determined using the same methods [22]. Z-score values <−2.0 and >2.0 were considered abnormal. Ventricular systolic function impairment was recognized when EF <50% [18].

Biventricular myocardial deformation was evaluated using a dedicated CVi42 tissue tracking module. In the end-diastolic phase in all slices that encompassed the ventricles, LV endocardial and epicardial as well as RV endocardial contours were automatically traced by the software and manually corrected, excluding papillary muscles and trabeculations. In the same phase, RV free wall epicardial contours were traced manually. After propagation of the contours through the other phases, they were assessed and corrected if necessary. LV and RV global circumferential and global radial strain (LV/RV GCS and LV/RV GRS) were assessed using the cine short-axis slices. The global longitudinal strain was measured on the two- and four-chamber cine images for the LV (LV GLS) and using the four-chamber view for the RV (RV GLS).

The studies were assessed for the presence of late gadolinium enhancement (LGE), with LV divided into segments using the 17-segment cardiac model. Increased signal intensity in the late scans was considered as LGE when it was visible in two different spatial orientations. Besides categorical differentiation between patients with and without features of myocardial fibrosis, the extent of LGE was quantitatively assessed using a dedicated module, with pathological enhancement defined as myocardium with signal intensity >6 SD above the mean in a reference region of interest in an effectively nulled part of the myocardium [25].

### 2.4. Statistical Analysis

Statistical analysis was performed using R Studio (NHST statistics) with dabest package version 0.3.1 [26]. Continuous variables were checked for normal distribution with both the Shapiro–Wilk test and histograms’ assessment and are presented as mean ± SD if normally distributed or as median (interquartile range) if non-normally distributed. The variables were compared between the LVNC patients and the age- and sex-matched controls using the paired Student’s *t*-test if normally distributed or the Wilcoxon signed-rank test if non-normally distributed. Categorical variables are reported as count (percentage) and were compared between the groups using the McNemar test.

The comparison between LVNC patients with LGE and those without LGE was performed using the unpaired Student’s *t*-test or the Mann–Whitney depending on the normality of distribution; categorical variables were compared using the chi-square test.

To further explore the data, in addition to the classic null hypothesis significance testing, we used permutation tests, the results of which are presented in separate columns of the tables. The effect size of the difference between patients and healthy controls was analyzed using the paired mean difference with bias-corrected and accelerated 95% confidence intervals obtained with bootstrapping on 5000 resamples.

*p*-values of less than 0.05 were considered statistically significant.

## 3. Results

### 3.1. Patient Characteristics

The LVNC group consisted of 16 patients aged 6–17 years (mean age: 13.3 ± 3.2, 6 girls (38%)). Only 1 patient (6%) declared episodes of chest pain, whereas dyspnea or syncope was not reported. No patients had a history of thromboembolic events. A significant portion of the children with LVNC (5/16, 31%) had a family history of LVNC or other cardiomyopathies. In a majority of patients (9/16, 56%), pathologic molecular variants associated with LVNC were identified in the following genes: *PRDM16, RBM20* (in two patients), *ACTN2, HCN4, EYA4, KCNQ1, TTN, ACTC1, HCCS.* NYHA functional class was assessed as I in 25% of patients (4/16) and as II in the remaining 75% (12/16) of children. In half of the patients (8/16), abnormalities in resting ECG or 24 h monitoring were noted (bradycardia in 5 children, junctional rhythm in 2, negative T-wave in 2, partial right bundle block in 1, and ST depression in 1). Most children (12/16, 75%) were treated pharmacologically and received between one and three medications (ACE inhibitor, beta-blocker, spironolactone, salbutamol, acetylsalicylic acid).

The patients were compared with a control group composed of age- and sex-matched children, who presented without any structural or functional cardiac abnormalities detected in CMR. The patients with LVNC did not differ significantly from the controls in terms of height, BMI, or BSA (Table 1). There were borderline differences in body weight between the study groups, with the average weight of children with LVNC being lower (46.3 ± 19.5 kg vs. 56.1 ± 22.0 kg, *p* = 0.055).

**Figure 1:**
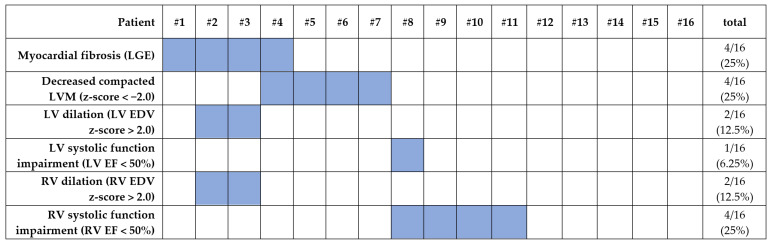
Prevalence of ventricular abnormalities in the LVNC group as detected in CMR.

CMR results in the study groups are presented in Table 2, whereas the prevalence of the main abnormalities in the LVNC group is demonstrated in Figure 1.

### 3.2. Left Ventricle

**Figure 2:**
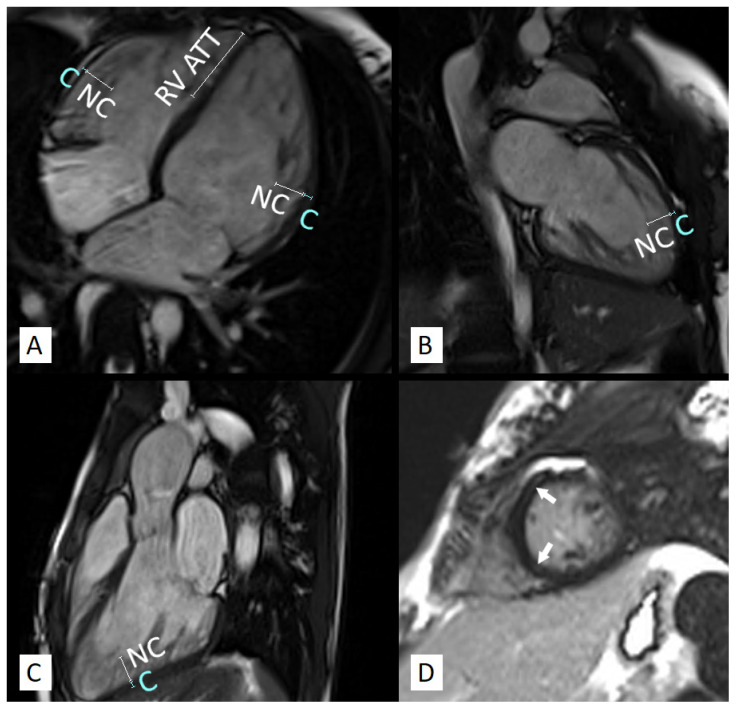
CMR study of a 17-year-old girl diagnosed with LVNC. The patient presented with enlarged left and right ventricles with preserved systolic function and increased trabeculations of both ventricles (**A**–**C**). RV apical trabecular thickness (ATT) and LV and RV noncompacted (NC) and compacted (C) layer measurements are marked on the long-axis views (**A**–**C**). Mid-wall late gadolinium enhancement is visible in the mid-ventricular septal segments (**D**, arrows).

As expected, the patients with LVNC had significantly higher maximal LV NC/C ratio (3.30 (2.46, 3.75) vs. 0.89 (0.46, 1.15), *p* < 0.001), trabeculated LVMI (25.0 ± 11.1 g/m^2^ vs. 4.8 ± 3.3 g/m^2^, *p* < 0.001) and trabeculated/global LVM ratio (31.3 ± 7.9% vs. 8.3 ± 5.3 g/m^2^, *p* < 0.001) compared to the control group (Figure 2). Compacted LVMI was comparable between the groups.

**Figure 3:**
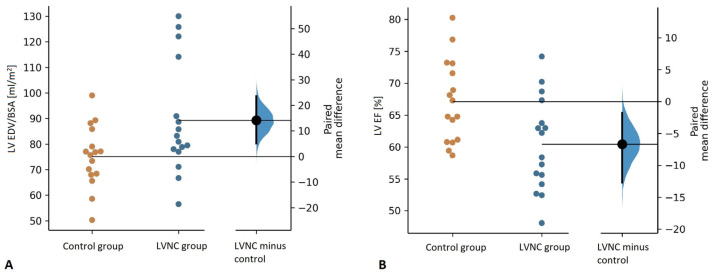
Comparison of the left ventricular end-diastolic volume indexed for body surface area (LV EDV/BSA) (**A**) and the left ventricular ejection fraction (LV EF) (**B**) between the control and the LVNC group.

Children with LVNC had higher mean EDV/BSA than the controls (82.1 mL/m^2^ (77.5, 102.5) vs. 76.3 mL/m^2^ (68.2, 82.4), *p* = 0.015) (Figure 3a). In the LVNC group, 2 patients (12.5%, 1 girl) presented with LV enlargement, with both of them having LGE and RV enlargement while retaining preserved LV systolic function (Figure 1).

Mean LV EF was significantly lower in children with LVNC than in the controls (60.4 ± 7.3% vs. 67.1 ± 6.5%, *p* = 0.037) (Figure 3b). Nevertheless, the mean LV EF value in the LVNC group was substantially higher than the cut-off value for preserved LV systolic function, and only 1 patient, an 8-year-old boy, presented with mild LV systolic function impairment (LV EF = 48%) [27].

**Figure 4:**
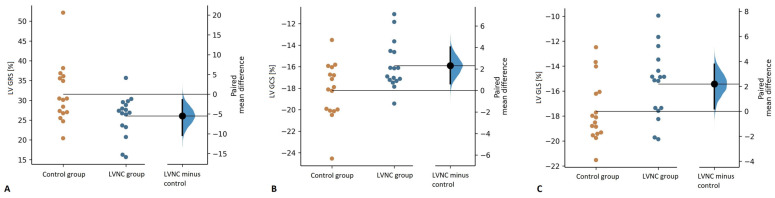
Comparison of left ventricular global radial strain (GRS) (**A**), global circumferential strain (LV GCS) (**B**), and global longitudinal strain (LV GLS) (**C**) between the control and the LVNC group.

Feature tracking analysis revealed impaired LV GRS, GCS, and GLS in the LVNC group compared to the control group (LV GRS: 26.0 ± 5.2% vs. 31.6 ± 7.4%, *p* = 0.033; LV GCS: -15.9 ± 2.2% vs. -18.2 ± 6.8%, *p* = 0.022; GLS: -15.4 ± 2.8% vs. -17.6 ± 2.5%, *p* = 0.032) (Figure 4).

### 3.3. Right Ventricle

**Figure 5:**
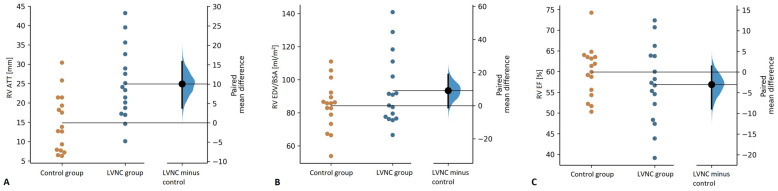
Comparison of right ventricular apical trabeculation thickness (RV ATT) (**A**), end-diastolic volume indexed for body surface area (RV EDV/BSA) (**B**), and ejection fraction (RV EF) (**C**) between the control and the LVNC group.

The study groups did not differ in RV NC/C ratio; however, LVNC patients had significantly thicker RV apical trabeculations (RV ATT: 24.9 ± 9.2 mm vs. 14.9 ± 7.4 mm, *p* = 0.006) (Figure 5a). LVNC patients did not differ from the controls in RV EDV/BSA (Figure 5b), yet RV enlargement was noted in 2 patients, in whom LV was also dilated.

RV EF values were comparable between the study groups (Figure 5c); however, in 4 LVNC patients, RV EF was <50%. Additionally, children with LVNC presented with impaired RV GRS and GCS compared to the controls (17.1 ± 6.7% vs. 23.0 ± 8.7%, *p* = 0.004 and −10.3 ± 4.6% vs. −12.9 ± 3.3%, *p* = 0.015, respectively), with no differences in mean RV GLS values.

### 3.4. LGE in Children with LVNC

In the LVNC group, mid-wall left ventricular LGE was observed in 4 of the 16 patients (25%). In this subgroup of children with LVNC, mean LGE percentage of LV mass was 5.6 ± 2.2% and involved between 1 and 4 LV segments. Children with LGE did not differ from the other participants with diagnosed LVNC in terms of age or anthropometric parameters (Table 3). They had significantly lower HR during CMR studies and all 4 had bradycardia in ECG monitoring (4/4, 100% vs. 1/12, 8%; *p* < 0.001).

LV NC/C ratio, LV EF, and LV global strain values did not differ between the two groups. Yet compared to other children with LVNC, patients with LGE had higher LV EDV/BSA (123.0 ± 6.7 mL/m^2^ vs. 78.1 ± 9.6 mL/m^2^, *p* < 0.001) and compacted LVMI (67.9 ± 6.1 g/m^2^ vs. 48.0 ± 5.5 g/m^2^, *p* < 0.001).

Children with LGE had more pronounced RV trabeculations (RV ATT: 36.8 ± 6.1 mm vs. 21.0 ± 6.1 mm, *p* < 0.001; RV NC/C: 5.48 ± 1.15 vs. 2.90 ± 0.74, *p* < 0.001) compared to the other patients with LVNC. They also presented with higher RV EDV/BSA (124.7 ± 13.0 mL/m^2^ vs. 83.0 ± 9.7 mL/m^2^, *p* < 0.001). RV EF and RV global strain values did not differ significantly between the two subgroups.

## 4. Discussion

LVNC cardiomyopathy is the third most common primary cardiomyopathy in the pediatric population, with the highest incidence observed in infants [2]. In order to investigate LV and RV morphological and structural abnormalities in children and adolescents with LVNC, we conducted a case-control study involving a group of 16 pediatric patients with the cardiomyopathy confirmed in CMR according to the Jacquier criteria [21]. The majority of the children had a family history of LVNC or other cardiomyopathy and/or had a genetic mutation associated with the condition. Most of the patients presented with NYHA II functional class and a half had ECG abnormalities, most commonly bradyarrhythmias. Simultaneously, the patients scarcely reported clinical symptoms, and no history of confirmed thromboembolic events was noted.

The main findings of this case-control study are that children with LVNC have:

- Dilated LV, decreased LVEF, and impaired LV myocardial deformation;
- Increased RV apical trabeculations and decreased RV myocardial deformation, while RV EF is preserved.

Additionally, we found that among pediatric patients with LVNC those with LGE had significantly higher LV and RV EDV indexed for BSA and increased RV trabeculations.

### 4.1. LV Morphology and Function

In our study, as expected by the definition of the cardiomyopathy, we observed markedly increased LV trabeculations in the LVNC group, which was indicated by higher NC/C ratio, trabecular LVMI, and trabecular to global myocardial mass ratio. Importantly, the LVNC group was characterized by significantly lower, yet still preserved, mean LV EF (60 ± 7%) and substantially higher LV EDV/BSA compared to the control group (Figure 3). Moreover, 13% of patients presented with dilated LV, 6% with LV systolic function impairment, and 25% with LGE (Figure 2). Previously, in a cohort of 40 children with isolated LVNC diagnosed according to the Petersen criteria, Cheng et al. observed drastically impaired LV EF (mean 38 ± 17%) and markedly elevated indexed LV EDV (131 ± 56 mL/m^2^) [19]. In another CMR study, Uribe et al. observed relatively low mean LV EF in a group of 15 children with LVNC (51.7 ± 10.9%), with a third of patients presenting with LV EF <50% [18]. Compared to our study group, the results of those studies indicate more severe LV functional abnormalities in some pediatric cohorts, which can possibly be due to the participation of infants and toddlers, who have significantly worse prognoses [2,18,19]. The results of the only other pediatric case-control CMR study demonstrated findings similar to ours, with mean LV EF in the LVNC group significantly above the 50% threshold (64 ± 8%) [20]. Nevertheless, as opposed to our findings and the observations in adults, Nucifora et al. did not observe differences in LV size or systolic function between children with LVNC and the control group, possibly due to a considerably smaller group size [20,28].

The results of our study also indicate that children with LVNC have impaired LV myocardial deformation (Figure 4). It is an important finding as LV EF within the normal range has limited prognostic performance [29], whereas CMR-derived LV global strain was previously shown to be a powerful predictor of mortality in patients with preserved ejection fraction, independent and incremental to other imaging and clinical risk factors [30,31,32]. Our findings are congruous with the observations made by Nucifora et al., who also noted impaired LV global strain in children with LVNC. Additionally, decreased strain in pediatric LVNC was also demonstrated in echocardiographic studies [33,34,35].

More studies were conducted in adults, also yielding similar results. In a CMR study, 59 adults with LVNC had impaired LV deformation compared to healthy controls [36]. Importantly, there remained observable differences in GRS and GCS, also between the subgroup of LVNC patients with normal LV volumetrics and the controls [36]. Gastl et al. also demonstrated global strain impairment in adults with LVNC, additionally showing that the differences between affected individuals and healthy controls increase from LV base to the apex, which mirrors the usual distribution of increased trabeculations [37]. Importantly, even though longitudinal strain may be normal at the base, circumferential strain and strain rates were impaired in those segments [37]. Interestingly, LV GLS is not consistently found to be impaired in LVNC patients with preserved LV systolic function. Pu et al. showed significant differences in this parameter between the patients and the controls, comparably to the observations made by Nucifora et al. and the results of our study, in which 15/16 had preserved LV EF [20,38]. Simultaneously, Szűcs et al. and Gastl et al. did not observe differences in LV GLS between LVNC patients with LV EF >50% and controls, and in the study by Dreisbach et al. there were no significant differences between adults with LVNC and normal LV volumes and the controls [28,36,37]. Development of pediatric normative values for strain parameters derived using feature-tracking in CMR studies may allow for differentiation between affected individuals and those with physiological hypertrabeculation as well as for better identification of children with LVNC who are at increased risk due to significantly impaired myocardial deformation.

### 4.2. RV Morphology and Function

CMR permits for a precise RV assessment and the results of our study demonstrate features of subclinical RV involvement in pediatric patients with LVNC, characterized by thicker RV apical trabeculations and impaired RV deformation. Increased RV trabeculations in children with LVNC was rarely reported and mainly in echocardiographic studies. Pignatelli et al. diagnosed RV non-compaction in echocardiography in 8 out of 36 pediatric patients with LVNC [14]. Increased RV trabeculations were also reported by Koh et al., who observed it in 2 out of 10 children with LVNC who underwent two-dimensional echocardiography [12]. However, neither one of the patients had isolated LVNC, as one presented with an associated Ebstein’s anomaly and atrial septum defect, whereas the other had previously undergone the Fontan procedure for congenitally corrected transposition of the great arteries (cc-TGA) with ventricular septum defect [12]. In the only other CMR study describing RV in children with LVNC, Ergul et al. identified features of RV apical noncompaction in 4 out of 24 patients (16%) which were missed on echocardiography [13].

More data on RV morphology in LVNC is available from studies with adult participants. In a cohort of 113 LVNC patients, Andreini et al. diagnosed RV noncompaction in 20 adults (18%), a similar percentage as in the pediatric study by Ergul et al. [13,17]. Two other studies with adults compared RV trabeculations between LVNC patients and healthy controls, indicating increased RV trabeculation in individuals with LVNC, similarly to our findings [10,11]. Stacey et al. observed greater RV apical trabecular thickness in LVNC patients than in the control group and a significant correlation between ATT and LV NC/C ratio [10]. Moreover, thicker RV ATT was also associated with lower RV EF [10]. Simultaneously, similarly to our findings, the comparison between the LVNC and the control groups did not reveal significant differences in the RV lateral wall NC/C ratio [10]. Recently, Stämpfli et al. also reported RV involvement in LVNC, which was characterized by increased RV NC/C ratio and higher RV trabeculated area and volume [11]. Importantly though, as in the previous studies, we observed a significant overlap in the extent of RV trabeculation between LVNC patients and controls (Figure 5a) [10]. Nevertheless, the quantification of RV trabeculation, which can be more reliably done in CMR, may become a factor in comprehensive disease assessment, especially considering the correlation between RV abnormalities and prognosis [10,39,40].

The results concerning RV remodeling and systolic dysfunction in LVNC patients are contradictory. Stämpfli et al. did not observe significant differences between the study groups in RV size or systolic function, yet the latter was assessed by calculating fractional area change [11]. Similarly, a Swiss study comparing 20 patients with LVNC with a control group did not reveal differences between the groups in RV size or function [37]. In contrast, in adults with LVNC, Stacey et al. noticed increased RV EDV and significantly lowered RV EF (RV EF 35 ± 18%, assessed using the modified Simpson’s technique) [10]. In a large cohort of adults with LVNC, Vaidya et al. diagnosed on echocardiography RV enlargement and dysfunction in 26% and 27% of patients, respectively [40], whereas Leung et al. in a CMR study observed RV dysfunction (defined as RV EF <35%) in 7 out of 14 LVNC patients [39]. Additionally, Ashrith et al. showed mean RV EF of 44 ± 14% in a group of 42 adults with LVNC, with significantly lower RV systolic function in patients with LGE observed in LV myocardium [41]. In our study, the LVNC group did not differ from the control group in RV size or systolic function; however, 13% of the participants had RV enlargement, and 25% presented with impaired RV systolic function. Additionally, we observed features of impaired RV GCS and GRS in children with LVNC, which could suggest subclinical RV functional abnormalities in these patients and might be an early manifestation of overt RV anomalies observable in some adults. Future studies should investigate RV strain in larger groups of LVNC patients, including individuals without preserved LV EF.

### 4.3. LV Fibrosis in Pediatric LVNC

Myocardial fibrosis, as indicated by the presence of LGE, is an important, independent prognostic factor in cardiomyopathies, including LVNC [42,43,44]. A meta-analysis of 4 studies with an average follow-up duration of 5 years showed an almost 5-fold increase in cardiovascular events and an almost 10-fold increase in cardiac death in LVNC patients with LGE compared to other patients with LVNC, independent of LV EF [44]. In adults, myocardial fibrosis was also shown to correlate with disease severity, as the presence and extent of LGE were related to the extent of LV EF impairment and the severity of clinical symptoms [45]. A single pediatric study with 40 participants revealed significantly more common adverse events in patients with LVNC and myocardial fibrosis—2 out of 10 children underwent heart transplantation and 4 died, compared to 2 deaths among 30 participants without LGE [19].

In our study, LGE was noted in 25% of patients with LVNC, which is similar to data reported by Cheng et al. and Nucifora et al., who demonstrated the same prevalence of LGE in their cohorts [19,20]. The percentage of adults with LVNC in whom LGE is recorded varies; however, typically it is more commonly observed and the reported prevalence ranges between 13 and 55% [17,45,46,47,48,49]. The distribution of LGE is diverse—in the majority of patients it is mid-myocardial; however, subendocardial, subepicardial, and transmural LGE is also described. Most often it is observed in the interventricular septum. Interestingly, LGE, like deformation impairment, is not restricted to noncompacted regions, but also occurs in apparently normal segments, indicating that LVNC is a generalized cardiomyopathy [19,46,50]. The exact pathophysiology of myocardial fibrosis in LVNC has not been determined and it is not facilitated by the heterogeneous LGE distribution [19,46]. Subendocardial and transmural LGE may be explained by coronary artery thromboembolism [46,51] or coronary microcirculatory dysfunction, which manifests as decreased coronary flow reserve and was observed both in compacted and noncompacted segments [52]. Additionally, in children with LVNC, decreased myocardial perfusion and decreased flow reserve in noncompacted segments were observed in a positron emission tomography study [53]. Myocardial fibrosis may also be attributable to increased ventricular wall stress causing LV remodeling, as observed in dilated cardiomyopathy [54].

In the present study, we observed significant differences between the patients with LGE and the remaining children with LVNC. Similarly to the results of a larger pediatric study by Cheng et al., individuals with LGE had significantly larger LV, indicative of maladaptive remodeling and suggesting an association between myocardial fibrosis and remodeling [19]. Furthermore, we observed increased RV volume in patients with LGE, emphasizing RV involvement in the cardiomyopathy. Additionally, among children with myocardial fibrosis there were increased RV trabeculations, possibly indicating more generalized cardiac abnormalities. Interestingly, there was a significantly higher prevalence of bradyarrhythmias among children with LGE compared to other LVNC patients, with further studies on larger groups needed to investigate this observation. As opposed to the results of Cheng et al. and some of the studies conducted in adults, however, we did not observe differences in LV EF between patients with and without LGE, possibly due to the limited size of our study group [19,41,45,50].

### 4.4. Study Limitations

The main limitation of the study is the small size of the study group, which is related to the low LVNC prevalence in the pediatric population. The use of estimation statistics allowed us to approximate the effect size of the differences between groups and to a degree attenuated the impact of a small sample size. Nevertheless, the results of the study should be considered as hypothesis-generating and should be confirmed by larger, multicenter, prospective studies. Another limitation of this study is the fact that it was performed in a single tertiary center and hence the patients may not be representative of the general population. Thirdly, the study lacks longitudinal assessment of the patients. The evaluation of the clinical course of LVNC in children is much needed and requires further investigation. Future studies could focus on CMR features impacting prognosis and hence permitting better identification of individuals with increased cardiovascular risk.

## 5. Conclusions

LVNC in children and adolescents is associated with left ventricular enlargement, reduced systolic function, and impaired myocardial deformation. Moreover, pediatric patients with LVNC have increased right ventricular apical trabeculations and subclinical right ventricular myocardial deformation impairment. Left ventricular fibrosis is prevalent in a significant percentage of children with LVNC and is linked to biventricular remodeling.

## Figures and Tables

**Table 1:** Patient characteristics. BSA indicates body surface area; BMI, body mass index; HR, heart rate.

	LVNC (*n* = 16)	Control Group (*n* = 16)	*p*-Value	*p*-Value Permutation	Cohen’s D Effect Size (95% CI)
**Female sex**	6/16 (38%)	6/16 (38%)	n.s.	-	-
**Age [years]**	13.3 ± 3.2	13.3 ± 3.4	n.s.	n.s.	−0.02 (−0.10–0.05)
**Height [cm]**	153.6 ± 24.0	160.4 ± 19.4	n.s.	n.s.	−0.31 (−0.88–0.05)
**Weight [kg]**	46.3 ± 19.5	56.1 ± 22.0	n.s.	0.046	−0.47 (−0.94–−0.03)
**BSA [m^2^]**	1.40 ± 0.40	1.57 ± 0.39	n.s.	n.s.	−0.42 (−0.94–−0.05)
**BMI (kg/m^2^)**	18.5 ± 3.8	20.9 ± 4.8	n.s.	n.s.	−0.54 (−1.07–0.13)
**HR [min^−1^]**	64.7 (57.8, 78.2)	70.0 (66.9, 86.8)	n.s.	n.s.	−0.44 (−1.07–0.46)

**Table 2:** Comparison of left and right ventricular morphology and function assessed in CMR between patients with LVNC and the control group. LGE indicates late gadolinium enhancement; LV, left ventricle; NC/C, noncompacted to compacted ratio; LVMI, left ventricular mass index; LVM, left ventricular mass; EDV, end-diastolic volume; BSA, body surface area; EF, ejection fraction; GRS, global radial strain; GCS, global circumferential strain; GLS, global longitudinal strain; RV, right ventricle; ATT, apical trabeculation thickness.

	LVNC (*n* = 16)	Control Group (*n* = 16)	*p*-Value	*p*-Value Permutation	Cohen’s D Effect Size (95% CI)
**Left ventricle**					
**LGE**	4/16 (25%)	-	-	-	-
**LV NC/C**	3.30 (2.46, 3.75)	0.89 (0.46, 1.15)	<0.001	<0.001	3.12 (2.37–3.92)
**compacted LVMI [g/m^2^]**	53.0 ± 10.4	53.4 ± 10.6	n.s.	n.s.	−0.04 (−0.63–0.53)
**trabeculated LVMI (g/m^2^)**	25.0 ± 11.1	4.8 ± 3.3	<0.001	<0.001	2.48 (1.62–3.83)
**trabeculated/global LVM ratio (%)**	31.3 ± 7.9	8.3 ± 5.3	<0.001	<0.001	3.43 (2.72–4.29)
**LV EDV/BSA [mL/m^2^]**	82.1 (77.5, 102.5)	76.3 (68.2, 82.4)	0.015	0.008	0.80 (0.23–1.25)
**LV EF [%]**	60.4 ± 7.3	67.1 ± 6.5	0.037	0.034	−0.97 (−1.79–−0.22)
**LV GRS [%]**	26.0 ± 5.2	31.6 ± 7.4	0.033	0.028	−0.86 (−1.44–−0.06)
**LV GCS [%]**	−15.9 ± 2.2	−18.2 ± 6.8	0.022	0.018	0.95 (0.10–1.57)
**LV GLS [%]**	−15.4 ± 2.8	−17.6 ± 2.5	0.032	0.035	0.83 (−0.01–1.62)
**Right ventricle**					
**RV NC/C**	3.55 ± 1.41	3.12 ± 1.09	n.s.	n.s.	0.34 (−0.44–1.10)
**RV ATT [mm]**	24.9 ± 9.2	14.9 ± 7.4	0.006	0.004	1.20 (0.34–1.96)
**RV EDV/BSA [mL/m^2^]**	93.4 ± 21.2	84.3 ± 14.7	n.s.	n.s.	0.50 (−0.14–0.98)
**RV EF [%]**	56.9 ± 9.3	59.9 ± 6.1	n.s.	n.s.	−0.39 (−1.09–0.25)
**RV GRS [%]**	17.1 ± 6.7	23.0 ± 8.7	0.004	0.003	−0.77 (−1.23–−0.33)
**RV GCS [%]**	−10.3 ± 4.6	−12.9 ± 3.3	0.015	0.013	0.64 (0.21–1.17)
**RV GLS [%]**	−18.3 ± 4.7	−16.22 ± 5.7	n.s.	n.s.	−0.40 (−1.20–0.41)

**Table 3:** Comparison between LVNC patients with and without myocardial late gadolinium enhancement (LGE) indicative of fibrosis. BSA indicates body surface area; BMI, body mass index; HR, heart rate; LGE, late gadolinium enhancement; LV, left ventricle; LVM, left ventricular mass; LVMI, left ventricular mass index; EDV, end-diastolic volume; EF, ejection fraction; GRS, global radial strain; GCS, global circumferential strain; GLS, global longitudinal strain; RV, right ventricle; ATT, apical trabeculation thickness; NC/C, noncompacted to compacted ratio.

	LVNC with LGE (*n* = 4)	LVNC without LGE (*n* = 12)	*p*-Value	*p*-Value Permutation	Cohen’s D Effect Size (95% CI)
**Female sex**	1/4 (25%)	5/12 (42%)	n.s.	-	-
**Age [years]**	15.4 ± 2.4	12.6 ± 3.2	n.s.	n.s.	0.95 (−0.44–1.73)
**Height [cm]**	156.8 ± 24.1	152.6 ± 25.0	n.s.	n.s.	−0.04 (−1.24–1.07)
**Weight [kg]**	45.8 ± 18.4	46.5 ± 20.7	n.s.	n.s.	0.17 (−1.48–1.11)
**BSA [m^2^]**	1.4 ± 0.4	1.4 ± 0.4	n.s.	n.s.	0.05 (−1.26–1.12)
**BMI (kg/m^2^)**	18.1 ± 3.4	18.7 ± 4.1	n.s.	n.s.	−0.16 (−1.50–0.68)
**HR [min^−1^]**	47.6 (42.3, 58.1)	71.5 (60.8, 83.2)	0.018	0.020	−1.46 (−2.35–−0.61)
**Left ventricle**					
**Number of segments with LGE**	2.0 ± 1.4	-	-	-	-
**LGE [% LV Mass]**	5.6 ± 2.2	-	-	-	-
**LV NC/C**	3.50 (2.86, 3.82)	2.99 (2.45, 3,64)	n.s.	n.s.	0.03 (−0.96–1.00)
**compacted LVMI [g/m^2^]**	67.9 ± 6.1	48.0 ± 5.5	<0.001	<0.001	3.53 (2.21–4.96)
**trabeculated LVMI (g/m^2^)**	35.1 ± 17.1	21.6 ± 6.1	0.028	0.027	1.41 (−0.12–2.65)
**trabeculated/global LVM ratio (%)**	32.8 ± 9.4	30.8 ± 7.7	n.s.	n.s.	0.26 (−0.80–1.78)
**LV EDV/BSA [mL/m^2^]**	123.0 ± 6.7	78.1 ± 9.6	<0.001	<0.001	4.94 (3.29–6.86)
**LV EF [%]**	61.3 ± 7.1	60.1 ± 7.7	n.s.	n.s.	0.16 (−0.82–1.36)
**LV GRS [%]**	22.8 ± 7.9	27.1 ± 3.8	n.s.	n.s.	−0.88 (−3.39–1.07)
**LV GCS [%]**	−14.6 ± 3.6	−16.4 ± 1.5	n.s.	n.s.	0.83 (−1.19–3.68)
**LV GLS [%]**	−16.3 ± 3.4	−15.1 ± 2.7	n.s.	n.s.	−0.42 (−1.77–1.03)
**Right ventricle**					
**RV ATT [mm]**	36.8 ± 6.1	21.0 ± 6.1	<0.001	0.001	2.60 (1.06–3.98)
**RV NC/C**	5.48 ± 1.15	2.90 ± 0.74	<0.001	0.001	3.05 (1.12–4.68)
**RV EDV/BSA [mL/m^2^]**	124.7 ± 13.0	83.0 ± 9.7	<0.001	<0.001	3.99 (2.33–5.53)
**RV EF [%]**	61.2 ± 8.8	55.4 ± 9.4	n.s.	n.s.	0.63 (−0.37–1.80)
**RV GRS [%]**	16.0 ± 6.6	17.4 ± 7.1	n.s.	n.s.	−0.20 (−1.18–1.19)
**RV GCS [%]**	−9.6 ± 3.7	−10.5 ± 3.3	n.s.	n.s.	0.26 (−1.36–1.27)
**RV GLS [%]**	−18.1 ± 7.3	−18.3 ± 3.9	n.s.	n.s.	0.05 (−1.64–1.77)

## Data Availability

The data presented in this study are available upon reasonable request from the corresponding author.
